# Iron Deficiency and Acute Seizures: Results from Children Living in Rural Kenya and a Meta-Analysis

**DOI:** 10.1371/journal.pone.0014001

**Published:** 2010-11-16

**Authors:** Richard Idro, Samson Gwer, Thomas N. Williams, Tuda Otieno, Sophie Uyoga, Gregory Fegan, Piet A. Kager, Kathryn Maitland, Fenella Kirkham, Brian G. R. Neville, Charles R. J. Newton

**Affiliations:** 1 Department of Paediatrics and Child Health, Mulago Hospital/Makerere University, Kampala, Uganda; 2 Centre for Geographic Medicine Research – Coast, Kenya Medical Research Institute, Kilifi, Kenya; 3 Department of Paediatrics, John Radcliffe Hospital, Oxford, United Kingdom; 4 Infectious Diseases Epidemiology Unit, Department of Epidemiology and Population Health, London School of Hygiene and Tropical Medicine, London, United Kingdom; 5 Department of Infectious Diseases, Tropical Medicine and AIDS, Academic Medical Centre, Amsterdam, The Netherlands; 6 Department of Paediatrics and Wellcome Trust Centre for Clinical Tropical Medicine, Faculty of Medicine, Imperial College, London, United Kingdom; 7 Department of Paediatric Neurology, Southampton General Hospital, Southampton, United Kingdom; 8 Neurosciences Unit, The Wolfson Centre, University College London-Institute of Child Health, London, United Kingdom; 9 Clinical Research Unit, London School of Hygiene and Tropical Medicine, London, United Kingdom; Tulane University, United States of America

## Abstract

**Background:**

There are conflicting reports on whether iron deficiency changes susceptibility to seizures. We examined the hypothesis that iron deficiency is associated with an increased risk of acute seizures in children in a malaria endemic area.

**Methods:**

We recruited 133 children, aged 3–156 months, who presented to a district hospital on the Kenyan coast with acute seizures and frequency-matched these to children of similar ages but without seizures. We defined iron deficiency according to the presence of malarial infection and evidence of inflammation. In patients with malaria, we defined iron deficiency as plasma ferritin<30µg/ml if plasma C-reactive protein (CRP) was<50mg/ml or ferritin<273µg/ml if CRP≥50mg/ml, and in those without malaria, as ferritin<12µg/ml if CRP<10mg/ml or ferritin<30µg/ml if CRP≥10mg/ml. In addition, we performed a meta-analysis of case-control studies published in English between January 1966 and December 2009 and available through PUBMED that have examined the relationship between iron deficiency and febrile seizures in children.

**Results:**

In our Kenyan case control study, cases and controls were similar, except more cases reported past seizures. Malaria was associated with two-thirds of all seizures. Eighty one (30.5%) children had iron deficiency. Iron deficiency was neither associated with an increased risk of acute seizures (45/133[33.8%] cases were iron deficient compared to 36/133[27.1%] controls, *p* = 0.230) nor status epilepticus and it did not affect seizure semiology. Similar results were obtained when children with malaria, known to cause acute symptomatic seizures in addition to febrile seizures were excluded. However, in a meta-analysis that combined all eight case-control studies that have examined the association between iron deficiency and acute/febrile seizures to-date, iron deficiency, described in 310/1,018(30.5%) cases and in 230/1,049(21.9%) controls, was associated with a significantly increased risk of seizures, weighted OR 1.79(95%CI 1.03–3.09).

**Conclusions:**

Iron deficiency is not associated with an increased risk of all acute seizures in children but of febrile seizures. Further studies should examine mechanisms involved and the implications for public health.

## Introduction

Acute seizures are common in children admitted to district hospitals in sub-Saharan Africa over 10% of admissions may report seizures. Infections are the predominant precipitants and in malaria endemic areas, *Plasmodium falciparum* malaria has been described as the leading cause in children 6 months and older [1]. Three types are described: i) febrile seizures, relatively benign seizures precipitated by fever; b) acute symptomatic seizures, here in the context of children with acute central nervous system infections and, c) initial seizures in patients developing epilepsy [2].

Iron deficiency is also common in the same regions of Africa. Although the results are conflicting, some reports from other settings suggest an increased prevalence of febrile seizures in iron deficient children [3], [4], [5], [6], [7], [8], . Whether the high prevalence of iron deficiency in sub Saharan Africa contributes to the high incidence of acute seizures in this region has not been explored.

Iron is involved in several brain processes such as neuro-metabolism, myelination, and, neuro-transmitter function. The metabolism of several neuro-transmitters and monoamine and aldehyde oxidases is reduced in patients with iron deficiency (reviewed in [12], [13]). It has also been suggested that fever aggravates the negative effects of iron deficiency on the brain: that iron deficient children may have a lowered threshold for and an increased risk of febrile seizures and may also influence the type, duration or recurrence of seizures [3].

The diagnosis of iron deficiency is problematic in children with acute febrile illnesses and even more so, in those with *Plasmodium falciparum malaria*. A combination of tests; erythrocyte packed cell volume (PCV) and mean cell volume (MCV), serum iron, ferritin, transferrin saturation and soluble transferrin receptor levels (sTfR) and/or bone marrow aspirate staining are used. Levels of most of the biochemical markers are altered during an acute infection and may therefore be unreliable. Although sTfR is a sensitive indicator of iron status in adults [14] and is unaffected by most infectious and inflammatory conditions, it is of questionable value in children with malaria [15], [16], [17]. Even in the same study, different cut off values have sometimes been used in different countries because of this [17]. In recent studies, iron deficiency has therefore been defined by plasma ferritin levels but using high cut-off levels [18], [19]. This is even more pertinent because unlike sTfR, ferritin metabolism is not affected by the two common hemoglobinopathies in the region, α-thalassemia and the sickle cell trait [20].

As part of an epidemiological study examining infectious, biochemical and genetic risk factors for acute seizures in rural Africa [1], [21], [22], we recruited children hospitalized with acute seizures from a defined area in coastal Kenya and compared them to a similar group of children without seizures from the same area. This is a malaria endemic area where iron deficiency, defined as two of the following: (i) low ferritin, (ii) low iron and/or high transferrin in plasma, (iii) markedly reduced or absent iron in bone marrow aspirates and (iv) increase in hemoglobin level after iron supplementation occurs in 33%[23]. We have previously shown that α-thalassemia, a common red blood cell defect in the region that also presents with microcytic anemia is not associated with an increased risk of acute seizures [21]. Here, we examined the hypothesis that iron deficiency in children is associated with an increased risk of acute seizures. In view of the conflicting reports in previous studies, we also present a meta-analysis of published case-control studies that have examined the relationship between iron deficiency and acute seizures in general or febrile seizures specifically, to date.

## Methods

### Case Control Study

This was a case control study of children with acute seizures (cases), or age-matched controls without seizures, admitted to Kilifi District hospital, on the coast of Kenya.

### Ethics

Ethical approval for the study was granted by the Kenya Medical Research Institute and a written consent was obtained from parents or guardians of each participating child.

### Study area

Kilifi District Hospital serves a predominantly rural population and is the only hospital in the area where the majority of very sick children are admitted for treatment. Annually, it admits about 5,000 children under the age of 14 years. A demographic surveillance system that defines an area where the majority of patients attending the hospital live has been in place since 2001. This study area undergoes regular census three times a year. Each person has a unique identifier that individually links the census data to hospital admissions. Common diagnoses include pneumonia, malaria, severe malnutrition, diarrhea, and neonatal diseases. Lead poisoning is not a recognized problem in this rural area that has no industrial establishments.

### Participants

We estimated that at 80% power and 5% level of significance, with a ratio of cases to controls of 1∶1, a matched sample size of 132 cases and 132 controls could detect an odds ratio of 2.2. Cases were children from the surveillance area, aged 3–156 months, admitted to Kilifi District hospital with acute seizures during the presenting illness. Acute seizures were defined as reported or observed repeated rhythmic and involuntary muscle contractions or jerky movements of the limbs, face or mouth that was not stimulus sensitive. We chose to recruit all children with all acute seizures [24], rather than only febrile seizures as in previous studies because although malaria is a febrile illness, many seizures in *Plasmodium falciparum* malaria which is responsible for up to 2/3 of all acute seizures in children in the region, may be acute symptomatic rather than febrile seizures and making this distinction is difficult [25].

The next child to be admitted, of similar age to the case, from within the study area, with any diagnosis but no seizures, was recruited as a control to form the comparison group. Age matching within epochs of 3–12, 13–24, 25–36, 37–48, 49–60 and >60 months. Controls that developed seizures during hospitalization were renamed cases and another control recruited. Children with epilepsy (defined as 2 lifetime episodes of unprovoked seizures) and significant developmental delay were excluded.

### Recruitment procedures

#### i) Admission and Clinical Care

In Kilifi hospital, all children have standardized clinical and laboratory data prospectively collected on admission and at discharge or death and directly entered into a computer database. As part of a bigger epidemiological study on acute seizures, we modified this database to obtain additional data relevant to acute seizures [1].

On admission, the admitting clinician, usually a Medical or Clinical Officer, performed resuscitation and emergency care procedures such as correction of hypoxemia, hypoglycemia and hypovolemia for all cases and controls using the same standard guidelines [26], [27]. Parents were then asked for permission for their children to participate in the study, history was taken and physical examination performed. The history included number and a parental description of the types of seizures during the illness and past seizures. Patients received antimalarial and/or empiric antimicrobial therapy for malaria and bacterial infections. A change of antibiotics and the duration of treatment were guided by the results of blood culture, CSF analysis and diagnosis. Acyclovir was not routinely available.

After stabilization, children with iron deficiency were offered ferrous sulphate 20mg/kg/day for 30 days, and mebendazole, 100mg twice daily for 3 days. No follow up was however performed to determine whether this intervention was successful. The discharging clinician made the final diagnosis after review of the admission history, inpatient management notes and laboratory investigations. These diagnoses were checked by a supervising clinician and followed the current World Health Organization (WHO) guidelines for the management of common illnesses in hospitals with limited resources [27].

#### ii) Laboratory procedures

Venous blood was obtained for a full count (MDII; Beckman Coulter, Fullerton, CA), glucose (Analox Instruments, London, UK), blood smears for malaria parasites (Giemsa stained) and microbiological culture. Plasma from heparinized tubes was separated by centrifugation. Part of the plasma was used to determine levels of electrolytes and creatinine and the remaining immediately frozen together with the cell pellet and stored at −80°C. After completion of the study, plasma ferritin, iron and transferrin were assayed using Vitalab Selectra E, Clinical Chemistry Analyser (Vital Scientific NV, Dieren, The Netherlands). DNA was extracted from the cell pellet using PUREGENE® DNA extraction kit (GENTRA SYSTEMS, Boston MA) according to the manufacturers instructions and α-thalassemia genotyping for the common African 3.7kb α-globin deletion was performed employing PCR and gel electrophoresis methods described previously [28], [29].

#### iii) Definition of terms

- In children with malaria parasitemia, iron deficiency was defined as plasma ferritin<30µg/ml if CRP<50mg/ml or as ferritin<273µg/ml if CRP≥50mg/ml. In those without malaria parasitemia, iron deficiency was defined as plasma ferritin<12µg/ml in the absence of an inflammatory process (CRP<10mg/ml) or as ferritin<30µg/ml if CRP≥10mg/ml. The criterion offers a sensitivity of 75% and a specificity of 76%[18], [19].
- Anemia was defined as a hemoglobin concentration of <11g/dl. Microcytosis was defined as MCV below the age-corrected normal values for erythrocyte volumes (MCV of 70 fl/µl in children <2 years, of 73 fl/µl in children 2–4 years, 75 fl/µl in children 5–7 years and 76 fl/µl in children 8 years or older) [30].

### Data management and analysis

Individual clinical data was directly entered in a FileMaker 5.5 database at admission. Data was analyzed using Stata version 9.2 (Stata Corp, Tx). To examine the relationship between iron deficiency and acute seizures, we compared the proportions of cases and controls with iron deficiency using Pearson's chi square test. An analysis for trend was performed to examine a “dose effect” relationship between plasma ferritin level or proportion with iron deficiency and the number of seizures. In addition, the association between iron deficiency and the type and the proportion of patients with status epilepticus (effect on seizure duration) and outcome were assessed. Continuous measures that were approximately normally distributed data were compared using the unpaired student's t-test while markedly skewed data were compared using Wilcoxon's rank-sum test. A p-value of <0.05 was considered significant.

### Meta analysis of studies of iron deficiency and seizures in children

#### Inclusion criteria for studies

To be included in the meta-analysis, studies had to fulfill the following criteria:

- Published in English between January 1966 and December 2009
- Available through PUBMED
- Original study involving children
- Of case-control or comparative study design with clear comparative groups of cases with seizures and controls without seizures and
- A specified criteria defining iron deficiency in the study subjects. Where this information was inadequate, the details could be obtained from the authors.

#### Search strategy

**Figure 1:**
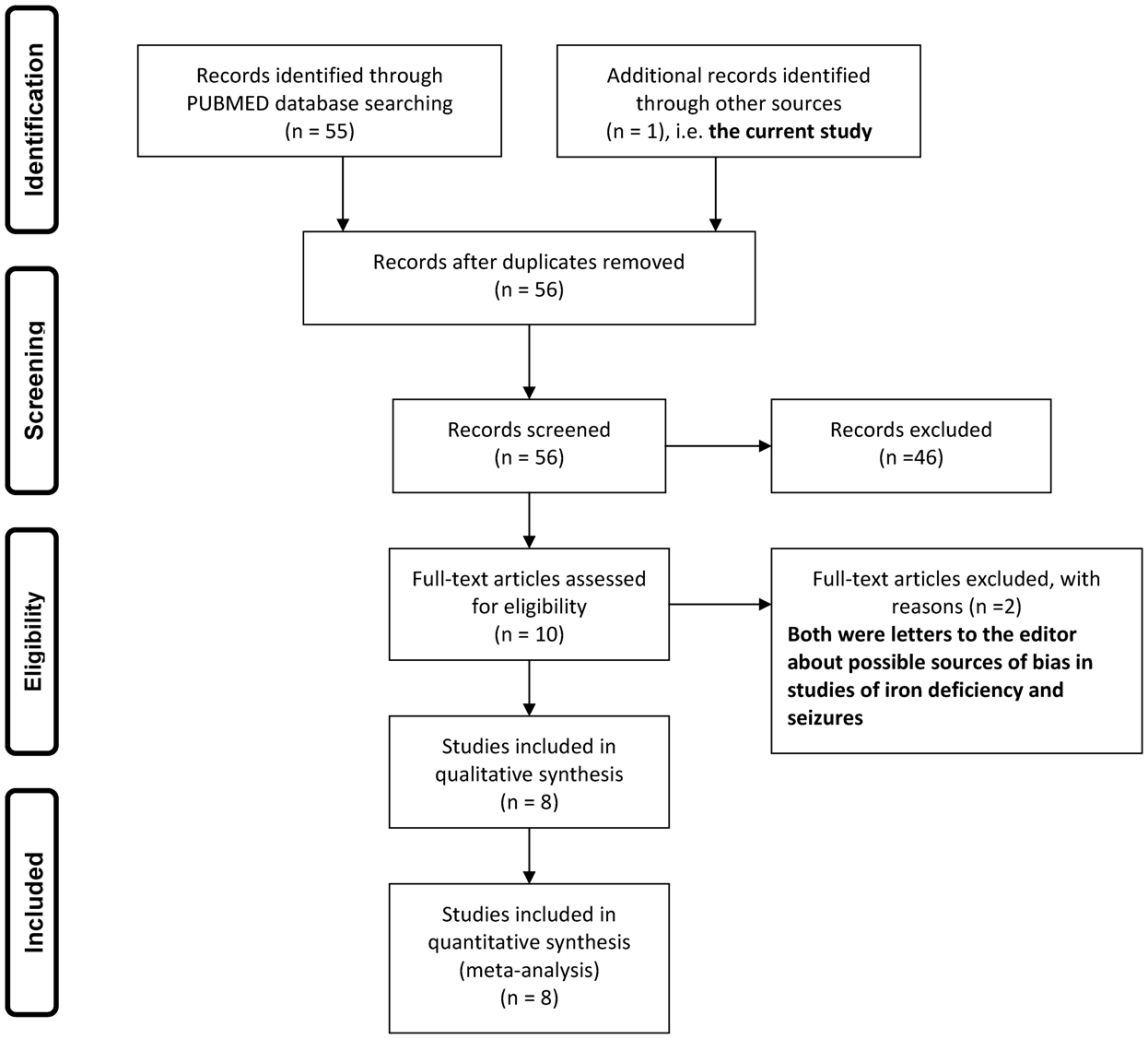
Study selection for the meta-analysis. Figure 1 is a flow diagram that shows the selection of studies for the meta-analysis investigating the association between iron deficiency and acute seizures in children.

Using the search phrase “iron deficiency” and “seizures” or “convulsions” for studies in PUBMED published between January 1966 and December 2009, we identified 55 publications. These were screened for eligibility and only 9 were selected, full texts of which were obtained [3], [4], [5], [6], [7], [8], [9], [10], [11], figure 1. Two of the nine papers were excluded because these were letters to the editor listing possible sources of bias in the earlier studies rather than original reports [10], [11]. The seven remaining studies [3], [4], [5], [6], [7], [8], [9] were included in the meta-analysis in addition to the current study.

#### Data extraction and management

Data from the eight studies was extracted into a standardized excel form and the results summarized together with the meta-analysis. The study variables included the total number of cases and controls in each study and the numbers and proportions in each group defined as being iron deficient. In addition, we obtained information on the definition used for iron deficiency. However, because the majority of studies used different tests or combination of tests to define iron deficiency, we neither did nor could we standardize the definition for iron deficiency. In one study [8], the published data contained inadequate information to answer all our questions and we contacted the corresponding author of the original study for clarification and for the original data. Data was extracted by one author (RI) and the accuracy checked by a second (SG).

The meta-analysis was also performed using STATA version 9.2. The risk ratios of the original studies are reported together with the combined estimate and 95% confidence intervals.

## Results

### The Kenyan Case Control Study

#### General characteristics of cases and controls

One hundred and thirty three children admitted to Kilifi District Hospital between January 2005 and January 2006 with acute seizures, were age-matched to the same number of hospital controls. The mean age, nutritional status and gender of cases and controls, were similar (table 1). Children with seizures were more likely to have had seizures in the past, were admitted after a shorter duration of illness, and had a higher admission temperature. Malaria was the commonest diagnosis in both cases and controls, and was associated with two thirds of all acute seizures. Other common diagnoses included respiratory tract infections and gastroenteritis.

**Table 1:** Characteristics of cases and controls on admission to hospital.

Clinical characteristics	Cases, N = 133	Controls, N = 133	*P* value
Mean (SD) age, months	30.3 (19.2)	28.8 (19.3)	0.510
Gender, male (%)	73 (54.9)	70 (52.6)	0.712
Median (IQR) duration of illness, days	2 (1–3)	3 (2–3)	0.047
Fever before hospitalization, (%)	129 (97.0)	107 (80.5)	<0.001
History of previous seizures, (%)	55 (41.4)	24 (18.1)	<0.001
Mean (SD) temperature on admission, °C	38.5 (1.2)	38.0 (1.2)	0.001
Malnutrition (weight for age Z score <−2)*	66 (49.6)	59 (44.4)	0.390
Diagnosis			
Malaria	89 (66.9)	42 (31.6)	<0.001
Respiratory tract infections	14 (10.5)	30 (22.6)	0.008
Gastroenteritis	8 (6.0)	12 (9.0)	0.352
Others	22 (17.3)	49 (36.8)	<0.001

*Using the 1978 World Health Organization standards.

#### Iron deficiency and seizures

A total of 81 (30.5%) children were iron deficient. Neither iron deficiency nor iron deficiency anemia was associated with acute seizures; 45/133 (33.8%) cases were iron deficient compared to 36/133 (27.1%) controls, Pearson's chi square 1.4378, *p* = 0.230, the mean and median levels of the biochemical markers of iron metabolism and the proportions of patients with iron deficiency and iron deficiency anemia were similar in cases and controls, table 2.

**Table 2:** Iron deficiency, α-thalassemia genotypes and acute seizures.

Indices	Cases, N = 133	Controls, N = 133	P-value
**Hematological indices**			
Mean (SD) white blood cell count/µl	13.0 (7.4)	14.1 (8.3)	0.240
Hemoglobin <110 g/L, (%)	115 (86.5)	117 (88.0)	0.713
Packed cell volume <33%	104 (78.2)	106 (79.7)	0.764
Mean cell volume (SD), fl	71.6 (9.4)	73.6 (12.1)	0.129
Microcytosis (age defined, %)*	86 (64.7)	72 (54.1)	0.080
**Biochemical indices of iron deficiency**			
Median (IQR) serum iron, µg/dl	16.9 (11.4–23.8)	18.7 (12.0–26.4)	0.135
Median (IQR) serum ferritin, µg/ml	126 (37–296)	106 (33–299)	0.479
Mean serum transferrin, mg/dl	223 (95)	227 (110)	0.720
Iron deficiency†	45 (33.8)	36 (27.1)	0.230
Iron deficiency anemia‡	41 (30.8)	30 (22.6)	0.127
**α-Thalassemia genotype**			0.269§
Normal (no deletion, %)	46 (34.6)	57 (42.9)	
Heterozygous (single deletion, %)	65 (48.9)	61 (45.9)	
Homozygous (double deletion, %)	22 (16.5)	15 (11.3)	

*Microcytosis is defined as age-corrected normal values (MCV<70 fl/µl in children <2 years, <73 fl/µl in children 2–4 years, <75 fl/µl in children 5–7 years and <76 fl/µl in children ≥8 years).

† In patients with malaria, iron deficiency was defined as plasma ferritin<30µg/ml if CRP<50mg/ml or as ferritin<273µg/ml if CRP≥50mg/ml and in those without malaria, it was defined as plasma ferritin<12µg/ml if CRP<10mg/ml or as ferritin<30µg/ml if CRP≥10mg/ml.

‡ Iron deficiency and hemoglobin <11g/dl.

§ Chi square test for trend.

We examined for a dose – response relationship between plasma ferritin or iron deficiency and the number of seizures the patient had during the illness. In either case, no dose response effect was observed (table 3). In addition, none of the hematological measures of iron deficiency (including MCV, PCV, ferritin and hemoglobin) was independently associated with seizures.

**Table 3:** Dose – Response relationship between plasma ferritin levels or the proportion of patients with iron deficiency and the number of seizures a child had during the acute illness.

Number of seizures	Linear Score	Number of patients	Median (IQR) plasma ferritin, µg/mL*	Number (%) with iron deficiency**
0 (Controls)	0	133	106 (33–299)	36 (27.1)
1	1	77	102 (29–299)	27 (35.1)
2	2	26	127 (56–210)	9 (34.6)
3	3	17	182 (62–303)	4 (23.5)
4 or more	4	13	219 (53–357)	3 (38.5)

*Test for linear trend; z = 1.46 and p = 0.143.

**Test for linear trend; z = 0.75 and p = 0.455.

Because malaria was over represented in cases in comparison to controls (2/3 of cases had malaria compared to 1/3 of controls), using a differential definition for iron deficiency as we did may introduce bias where the malaria specific definition is used more often in cases than in controls. We therefore performed a second analysis using a single definition for iron deficiency that disregarded the effects of malaria on ferritin levels but only stratified the ferritin levels by the inflammatory response, i.e. plasma ferritin<12µg/ml if CRP<10mg/ml or ferritin<30µg/ml if CRP≥10mg/ml. Such a definition led to an under ascertainment identifying only 43 children as iron deficient, yet, still, no association was observed between iron deficiency and acute seizures. Thus, only 20 (15%) cases had iron deficiency using the revised definition compared to 23 (17.3%) controls, Pearson's Chi Square 0.2497, p = 0.617.

All previous studies included only children thought to have febrile seizures. Because *Plasmodium falciparum malaria* is epileptogenic and some of the seizures in this infection may be acute symptomatic seizures rather than febrile seizures we performed a second analysis excluding all patients with malaria. Although the sample was small, even then, the proportions of patients with iron deficiency among cases and controls were similar; 13/44 (29.6%) of the remaining cases had iron deficiency compared to 22/91 (24.2%) of controls, p = 0.505.

We performed a conditional regression analysis to exclude possible confounding effects by nutritional status (weight for age), past seizures, fever and malaria, on the association between iron deficiency and acute seizures. Again, we found no independent association between iron deficiency and acute seizures. Instead, past seizures (adjusted OR 4.6 96% CI 2.2–9.9, p<0.001), fever (adjusted OR 12 96% CI 1.5–102, p = 0.021), and malaria (adjusted OR 6.1 96% CI 2.8–13.4, p<0.001) were independently associated with acute seizures.

To examine the effect of iron deficiency on seizure manifestation/semiology, we compared the type (focal or generalized), number (including recurrences after admission) and duration (proportion with status epilepticus) of seizures in cases with and without iron deficiency. There was no association between iron deficiency and seizure manifestation or recurrences in the ward. In addition, iron deficiency was not associated with an increased risk of status epilepticus. All children who presented with impaired consciousness or coma and all three who died were non iron-deficient, table 4.

**Table 4:** Iron deficiency and the number, type, duration and outcome of acute seizures in cases.

Clinical features	Cases with iron deficiency, n = 43	Cases without iron deficiency, n = 90	P value
Median (IQR) number of seizures	1 (1–2)	1 (1–2)	0.566
Seizure type, (%)*			0.396
Focal	6 (14.0)	18 (20.0)	
Generalized	37 (86.0)	72 (80.0)	
Status epilepticus, (%)	8 (10.1)	22 (11.8)	0.700
Level of consciousness, (%)			0.010†
BCS 0–2‡	0 (0)	7 (7.8)	
BCS 3–4	0 (0)	10 (11.1)	
BCS 5	43 (100.0)	73 (81.1)	
Outcome (death)	0 (0)	3 (1.6)	0.258

*Seizure manifestation described by parent.

† Using chi square test for trend.

‡ BCS = Blantyre Coma Score (Score 0–2 = coma, 3–4 impaired consciousness, 5 = full consciousness).

### Meta-analysis of Studies of Iron Deficiency and Seizures in Children

#### Qualitative analysis

All eight studies were hospital based and other than our study, only included children in the febrile seizure age group, 3 months – 6 years. However, each study used a different definition for iron deficiency (table 5) with different levels of ascertainment for iron deficiency. For example, in the Naples study, iron deficiency was defined using red cell indices and level of serum iron [3], plasma ferritin was used in the Jordan study [4] and iron deficiency was defined using zinc protoporphyrin level in the US study [5]. Despite the differences in ascertainment, iron deficiency was associated with an increased risk of febrile seizures in five of the eight studies [3], [4], [6], [8], [9]. However, of the three studies which did not show an increased risk of seizures in iron deficient subjects, only the Iranian study [7] did not appear to have a mixed phenotype. Thus, 5/26 cases in the US study had learning difficulties [5], and our report included all acute seizures.

**Table 5:** Studies of iron deficiency and seizures in children.

Study No.	Country and Region of study setting	Definition of iron deficiency (ID)	Cases, N	Cases with ID, n (%)	Controls, N	Controls with ID, n (%)	Ref
1	USA, North America	Free erythrocyte protoporphyrin >0.80 ng/L	23	2 (8.7)	25	10 (40.0)	[5]
2	Italy, Western Europe	Hb<10.5g/dl, MCV<70fl and serum iron<5.4µmol/L	146	44 (30.1)	146	21 (14.4)	[3]
3	Jordan, Middle East	Ferritin≤30µg/L	75	49 (65.3)	75	24 (32.0)	[4]
4	Pakistan, Asia	MCV<70fl	30	17 (56.7)	30	9 (30.0)	[8]
5	Canada, North America	MCV<70fl and RDW>15.6%	361	31 (8.6)	390	19 (4.9)	[6]
6	India, Asia	Ferritin ≤25µg/L	50	34 (68.0)	50	15 (30.0)	[9]
7	Iran, Middle East	Hb, HCT, MCV, MCHC, total RBC and serum iron <2 SD of normal value for age and TIBC>430 mcg/dl	200	88 (44.0)	200	96 (48.0)	[7]
8	Kenya, East Africa	In children with malaria, as ferritin<30µg/ml if CRP was <50mg/ml or ferritin<273µg/ml if CRP≥50mg/ml and if no malaria, as ferritin<12µg/ml if CRP<10mg/ml or ferritin<30µg/ml if CRP≥10mg/ml.	133	45 (30.8)	133	36 (27.1)	Current study
**Total**			**1,018**	**310 (30.5)**	**1,049**	**230 (21.9)**	

Abbreviations: CRP = C-reactive protein, Hb = Hemoglobin = HCT, hematocrit, ID = iron deficiency, MCV = mean cell volume, RBC = red blood cells, RDW = red blood cell distribution width, SD = standard deviation and TIBC = total iron binding capacity.

#### Quantitative analysis

**Figure 2:**
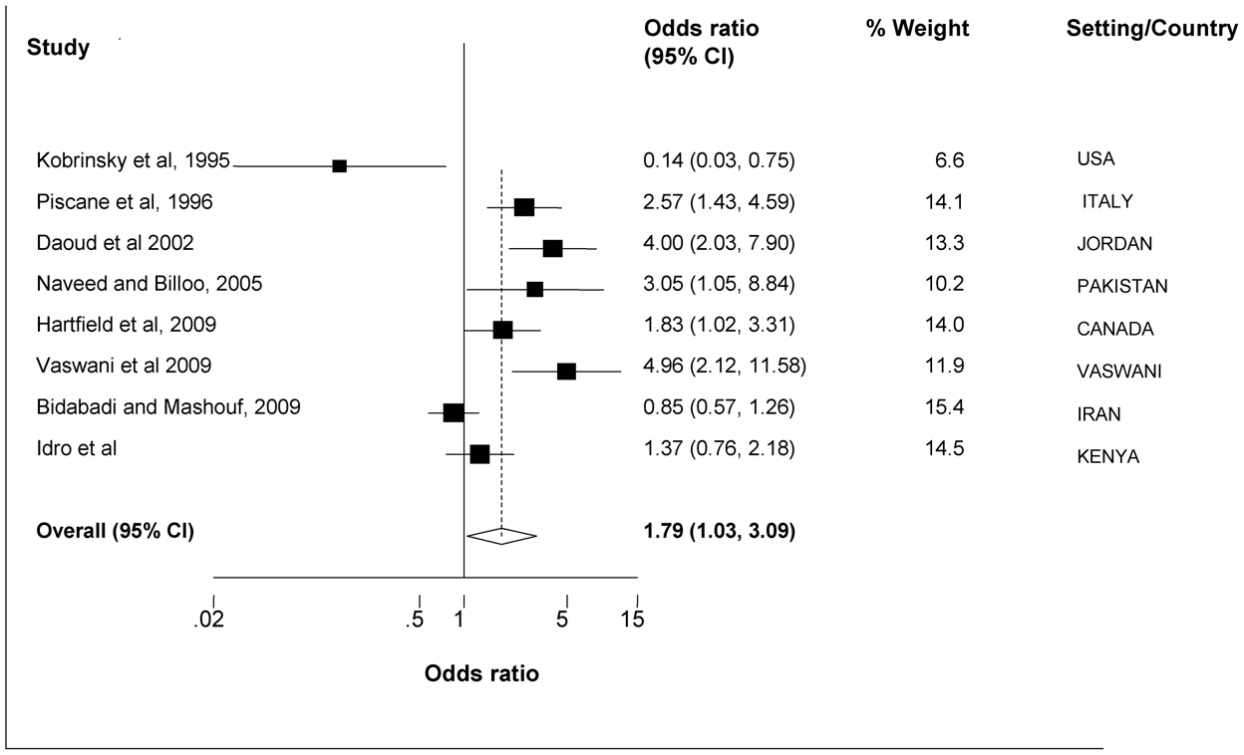
Meta-analysis of studies that have examined the relationship between iron deficiency and febrile seizures or acute seizures. Figure 2 is a forest plot of a meta-analysis of case control studies that have examined the relationship between iron deficiency and febrile seizures or acute seizures. The individual estimates and the relative contribution of the individual studies to the overall estimate are provided.

A total of 1,018 children with seizures and 1,049 without seizures were included in the analysis. Overall, iron deficiency was associated with an increased risk of seizures: iron deficiency was reported in 310(30.5%) cases and 230(21.9%) controls, weighted OR (random effects model) 1.79 (95% CI 1.03–3.09), Test for heterogeneity: Q = 37.397 on 7 degrees of freedom (p<0.001), I^2^>50% and moment-based estimate of between studies variance = 0.469, figure 2.

## Discussion

This study set out to examine the hypothesis that iron deficiency is associated with an increased risk of acute seizures in children. In those with seizures we further examined whether iron deficiency influences seizure manifestation, number or duration. We found no association between iron deficiency and acute seizures, and among cases, iron deficiency did not influence the manifestation or the number of acute seizures, the proportion with status epilepticus nor further recurrences in the ward. However, a meta-analysis of all eight case-control studies that have examined the relationship between febrile seizures or acute seizures and iron deficiency to-date suggested that iron deficiency may be associated with an increased risk of febrile seizures in children.

The relationship between iron deficiency and febrile seizures has been controversial. Most of the early and conflicting studies i) examined the relationship between iron deficiency and febrile seizures; ii) had small sample sizes and iii) used different markers and definitions for iron deficiency and iv) lead toxicity and celiac disease were also raised as possible confounding factors [10], [11]. More recently, three studies, two with relatively large sample sizes, re-examined the question but again with conflicting results. In the first study, 361 children with febrile seizures were compared to 390 controls; 15% of cases were iron deficient compared to 9% of controls [6]. In the second, 50 cases were compared to a similar number of controls; 68% of cases were iron deficient compared to 30% of controls [9]. An increased risk of seizures in iron deficient patients was, however, not observed in the third study of 200 cases and 200 matched controls in who, iron deficiency was less frequent in cases than controls [7].

A closer look at the studies which did not demonstrate an increase in seizures with iron deficiency reveals possible mixed phenotypes: other than the Iranian study [7], two of the three studies in which iron deficiency was not associated with seizures had mixed phenotypes. Thus, in the US study, 5/26 cases had learning difficulties [5] while our study included all acute seizures instead of only febrile seizures. In addition, our age range extended beyond the febrile seizure range. Although we performed a sub analysis in which we excluded patients with malaria – many of who may have acute symptomatic seizures - and included only patients with febrile seizure characteristics, even then, we observed no association between iron deficiency and seizures. The number of patients available for the sub analysis may however have been inadequate to answer the question.

Overall, these findings appear to demonstrate that iron deficiency is associated with an increased risk of febrile seizures specifically but not all acute seizures. Iron deficiency may be an important factor in the pathogenesis of febrile seizures but not all acute seizures in children with febrile illnesses. The use of different criteria for the diagnosis of iron deficiency is clearly a weakness and calls into question the use of a combined estimate in the meta-analysis. Despite this weakness, the pattern of results suggests a clear pattern of association between iron deficiency and febrile seizures but not necessarily so with all acute seizures. Neuro-developmental impairments secondary to iron deficiency may provide a background of abnormal neuron functioning and increase the risk of seizures with subsequent provoking events. Other factors e.g. the aetiological cause may be more important in acute symptomatic seizures.

As shown in an animal model, other possible mechanisms may include altered neuron excitability and neurotransmission [31]. In a study in which mice were fed on either an iron deficient or iron sufficient diet, with or without added lead, spontaneous seizures were more common in mice on the iron deficient diet with added lead. Mice on the iron deficient diet also had a shorter latency to onset of seizures and a longer mean duration of seizures and post-ictal period, suggesting that iron deficiency augments the effects of lead toxicity in mice [31]. Other animal studies have shown that iron deficiency affects myelination, and the enzymes, tyrosine and tryptophan hydroxylase, which are involved in the synthesis of neurotransmitters. Degradation of neurotransmitters is altered, and extracellular levels of noradrenaline and dopamine are elevated (reviewed in [12], [13]). In addition, the function of Thy-1, a cell adhesion molecule that plays a regulatory role in the release of neurotransmitters from vesicles, is altered [32]. Thy-1 deficiency may affect the release of neurotransmitters and synaptic efficacy, and could contribute to a variety of abnormal neuron-neuron communications.

Findings from the iron supplementation study in Tanzania that was terminated early because of increased all cause mortality and admissions to hospital [33], and the reduction in seizures in children with cerebral malaria after receiving an iron chelating agent as adjunct therapy [34] suggest that translating the findings of the meta-analysis into a widespread iron supplementation as a preventive measure in the community would have to be undertaken with care. In the Tanzanian study, children aged 1–35 months and living in the Islands of Pemba and Zanzibar were assigned to daily oral supplementation with a) iron and folic acid; b) iron, folic acid, and zinc; or c) placebo, to examine if universal supplementation with iron and folic acid in areas of high malaria transmission is safe. Those who received iron and folic acid with or without zinc were 12% (95% CI 2–23, p = 0.02) more likely to die or need treatment in hospital for an adverse event and 11% (1–23%, p = 0.03) more likely to be admitted to hospital [33]. On the other hand, in Zambia, deferoxamine 100 mg/kg/day infused for a total of 72 hours or placebo as adjunct therapy for cerebral malaria, was associated with a trend to increased mortality in the deferoxamine group (32/175, 18.3%) compared to those who received a placebo (19/177, 10.7%), adjusted odds ratio 1.8; 95% CI 0.9–3.6; *p* = 0.074 but a reduction in seizure recurrences during admission [34]. It should however be noted that both studies were conducted in areas with high prevalence of iron deficiency and high malaria transmission.

### Limitations

First, the population in this study was different from previous studies in that it was not limited to children with febrile seizures but included all children admitted with acute seizures associated with malaria which may not be febrile seizures [25], [35]. Second, because of the difficulty in defining iron deficiency in febrile children in malaria endemic areas [15], [16], we used a very strict definition with high ferritin cut-off levels. There was a trend towards higher frequency of microcytosis and lower MCV in children with seizures which, although may be confounded by alpha-thalassemia, suggests that our definition of iron deficiency may have been conservative. It is also possible that it is the microcytosis or hypochromia (i.e. reduced oxygen carrying capacity) which is associated with this trend, rather than the presence of iron deficiency. Other than malaria parasitemia, none of these markers was however independently associated with seizures.

Third, the use of only hospital controls in this study may have introduced a selection bias since these patients are more likely to have higher levels of iron deficiency than does the reference population. A better design would have included two sets of controls: hospital and community controls. Our study is however comparable to all the previous 7 case control studies that have examined the association between iron deficiency and seizures. All have only used hospital controls as the only comparative group. In this regard, we are comparing like to like. We also took additional measures to reduce such bias in our inclusion criteria namely, all controls came from the same geographical area as the cases (therefore had similar health risks) and were matched for age. More importantly, in an earlier survey of the same geographic area that aimed to establish hematological indices in children in the community and which defined iron deficiency using the same parameters as in the present case control study, we had established that 116/311 (37.3%) well children in this community were iron deficient, (Sammy Wambua, personal communication). A second study in the same area had documented similar results earlier [23]. It is therefore unlikely that not including community controls would have adversely affected our results. Instead, what is of more significance is the inclusion of all acute seizures (both symptomatic and febrile seizures) among the cases. This inclusion was in line with study objectives rather than bias: to explore any association between the high burden of acute seizures in children in this population and the high prevalence of iron deficiency.

Other possible bias includes recall and information bias (history of illness and seizures from parental report) and misclassification error resulting from definitions used for classifying iron deficiency. Fourth, unlike all the other studies included in the meta-analysis, subjects in our case control study had a very wide age range. Other than young infants (0–2 months old), we included all patients in the paediatric age group admitted to our children's wards (up to 13 years of age). It would have been more appropriate if only children 3months - 6 years were included. This not only makes comparison across studies difficult but also the aetiology of acute seizures may be different [1]. Fifth, in comparison to previous studies that examined the association between iron deficiency and febrile seizures in other regions, it is likely that children in this study had more severe iron deficiency. Genotyping for α-thalassaemia deletions allowed us to concurrently examine a prevalent genetic disorder that may affect iron metabolism and some attributes of the red cell. In the meta-analysis, the main source of bias was the different definitions used for iron deficiency. In addition, the inclusion of patients with mixed phenotypes in the different studies may have diluted the effect size.

### Conclusions

In conclusion, although our study found no association between iron deficiency and all acute seizures, an analysis of the individual studies and the combined result of case control studies to date suggest that iron deficiency is associated with an increased risk of febrile seizures in children. Further studies should examine the mechanisms involved, the possible association with subsequent epilepsy after severe malaria, and the implications for public health programs.
